# Reference Materials for Improving Reliability of Multiomics Profiling

**DOI:** 10.1007/s43657-023-00153-7

**Published:** 2024-03-06

**Authors:** Luyao Ren, Leming Shi, Yuanting Zheng

**Affiliations:** 1 State Key Laboratory of Genetic Engineering, School of Life Sciences and Human Phenome Institute, Fudan University, Shanghai, 200438 China; 2 Shanghai Cancer Center, Fudan University, Shanghai, 200032 China; 3 International Human Phenome Institutes, Shanghai, 200438 China

**Keywords:** Reference materials, Reference datasets, Quality control, Performance metrics, Reproducibility, Multiomics profiling, Molecular phenomics

## Abstract

High-throughput technologies for multiomics or molecular phenomics profiling have been extensively adopted in biomedical research and clinical applications, offering a more comprehensive understanding of biological processes and diseases. Omics reference materials play a pivotal role in ensuring the accuracy, reliability, and comparability of laboratory measurements and analyses. However, the current application of omics reference materials has revealed several issues, including inappropriate selection and underutilization, leading to inconsistencies across laboratories. This review aims to address these concerns by emphasizing the importance of well-characterized reference materials at each level of omics, encompassing (epi-)genomics, transcriptomics, proteomics, and metabolomics. By summarizing their characteristics, advantages, and limitations along with appropriate performance metrics pertinent to study purposes, we provide an overview of how omics reference materials can enhance data quality and data integration, thus fostering robust scientific investigations with omics technologies.

## Introduction

In recent years, the adoption of multiomics approaches in biomedical research and clinical application has increased significantly (Hasin et al. 2017; Hoadley et al. 2018). The integration of multiomics or molecular phenomics data (including genomics, epigenomics, transcriptomics, proteomics, and metabolomics) along with deep phenotypic data enables the discovery of correlations between the diverse levels of genetic and regulatory information and distinct phenotypic traits, fostering a more comprehensive understanding of biological processes and facilitating the identification of disease mechanisms, potential therapeutic targets, and disease biomarkers (Jiang et al. 2019; Mangiante et al. 2023; Martinez-Ruiz et al. 2023; Sammut et al. 2022). However, challenges exist in translating scientific research findings to clinical settings, particularly regarding the reproducibility of omics data (Bell et al. 2009; Foox et al. 2021; Khayat et al. 2021; Pan et al. 2022). The complexity of biological systems and potential technical artifacts from sample preparation, data generation and data analysis contribute to this challenge, which is further amplified in multiomics data integration (Krassowski et al. 2020). Implementing rigorous quality assurance (QA) and quality control (QC) measures is crucial to ensure the reliability of multiomics research (Bittremieux et al. 2018; Broadhurst et al. 2018; Zheng et al. 2022). QA involves processes and activities to prevent errors and ensure quality standards of the final product, whereas QC comprises activities to test and inspect the final product or service to meet quality standards (International Organization for Standardization, ISO 9000:^2015^).

Reference materials (RMs) are essential for both QA and QC in multiomics research (Broadhurst et al. 2018; Hardwick et al. 2017; Jennings et al. 2017; Lippa et al. 2022). RMs are well-characterized samples with known properties that can be used to validate the accuracy and reliability of analytical methods, assess the comparability of data generated by different laboratories or instruments, and serve as a standard against which the accuracy and precision of measurements can be evaluated (Bell et al. 2009; Foox et al. 2021; Hardwick et al. 2017). Although the terms “certified reference materials (CRMs)”, “standard reference materials (SRMs)”, “reference materials”, “reference standards”, “reference samples”, and “quality control samples” are often used with the same or very similar meaning related to the calibration and validation of analytical methods, there are important differences between them at the level of characterization, traceability, and certification that they offer. CRMs and SRMs are typically considered the most reliable and accurate standards for analytical measurements, while the others may have more limited or uncertain properties. According to ISO Guide 30:^2015^, a CRM is “a reference material, accompanied by a certificate, one or more of whose property values are certified by a procedure that establishes its traceability to an accurate realization of the unit in which the property values are expressed, and for which each certified value is accompanied by an uncertainty at a stated level of confidence” (International Organization for Standardization, ISO Guide 30:^2015^). In other words, a CRM is a reference material that has been thoroughly analyzed and certified by an authorized organization to have a known value for one or more properties, along with its associated uncertainty and a statement of metrological traceability. The certification process ensures that the material meets established international standards for accuracy and traceability. Official governing bodies, such as the National Institute of Standards and Technology (NIST) in the United States, the National Institute of Metrology (NIM) in China, and the European Commission's Joint Research Centre (JRC) in Europe, can provide certification for CRMs. Other accredited organizations can provide certification for CRMs as well. The term SRM is a specific term used by NIST for those meeting additional NIST-specific certification criteria in accordance with ISO Guide 31:2000 (National Institute of Standards and Technology 2023a). A RM is defined as “a material or substance one or more of whose property values are sufficiently homogenous and well established to be used for the calibration of an apparatus, the assessment of a measurement method, or for assigning values to materials” (International Organization for Standardization, ISO Guide 30:^2015^). RMs can also be referred to as “reference standards”, “reference samples”, or “quality control samples”. These materials can be prepared in-house or purchased from commercial suppliers. While RMs are generally considered to be of high quality, they may not have undergone the rigorous testing and certification required for CRMs.

Omics RMs mentioned in this review refer to well-characterized and validated samples used as quality control tools in various omics technologies. A major difference between traditional RMs and omics RMs is the number of properties values they encompass. Traditional RMs typically comprise a limited number of well-defined and characterized property values, often associated with physical and chemical attributes, placing a strong emphasis on traceability. In contrast, omics RMs encompass a significantly larger number of property values, reflecting the complex and diverse nature of biological omics data. It is important to note that there is currently no internationally recognized CRMs for massive analysis technologies, because current omics RMs do not fulfill the conventional criteria for established traceability of the assigned property values. The signals detected by omics technologies, such as DNA or RNA sequencing reads, mass spectrometry (MS) peaks, or nuclear magnetic resonance (NMR) spectra, cannot be directly traced back to the international system of units (SI units). While omics RMs may not have the same level of rigor as CRMs, they can still serve as a useful tool for quality control and method validation in omics research. Many ongoing efforts have been made to develop omics RMs. These RMs are typically prepared by reputable laboratories using standardized protocols and characterized for their stability, homogeneity, and variability, with their properties traceable to a reference measurement system. Examples of ongoing efforts to establish omics RMs include the Genome in a Bottle Consortium (GIAB) (Zook et al. 2014), the MicroArray/Sequencing Quality Control (MAQC/SEQC) (MAQC Consortium 2006; Fang et al. 2021; Jones et al. 2021), the External RNA Control Consortium (ERCC) (Baker et al. 2005), Clinical Proteomic Tumor Analysis Consortium (CPTAC) (Tabb et al. 2016), the Metabolomics Quality Assurance and Quality Control Consortium (mQACC) (Lippa et al. 2022), and the Chinese Quartet Project for multiomics profiling (Yang et al. 2023; Zheng et al. 2023).

RMs are widely recognized as essential for ensuring data quality in omics research; however, their current application is inadequate due to issues such as inappropriate selection of RMs for the intended purpose and a lack of understanding about when and how to use them (Begley and Ioannidis 2015; Bowden et al. 2018; Chiva et al. 2021; Evans et al. 2020; Zhang et al. 2020). This review aims to provide a comprehensive overview of currently available omics RMs for high-throughput technologies in omics research, with a focus on quality assessment across batches, platforms, and laboratories. We will first summarize RMs for each omics level (DNA, RNA, protein, and metabolite), including their intended usage, advantages and limitations (Table 1). We will then describe qualitative and quantitative properties of RMs that help determine accuracy and precision. Next, we will explain quality control metrics based on reference datasets or intrinsic relationships between reference sample groups. Finally, we will describe how to use RMs to improve biomarker discovery in omics studies and discuss considerations for utilizing appropriate RMs.

**Table 1:** Summary of reference materials for multiomics profiling

Omics	Category	Targeted molecule	Project/institute	Name	Sample source	Public availability of materials	References
DNA	Biological DNA	Whole-genome germline variants	GIAB	RM8398/RM8292/RM8393	gDNA from lymphoblastoid cell lines of a Utah woman with European Ancestry, an Ashkenazi Jewish trio and a Han Chinese trio	Yes	Chin et al. (2020), Wagner et al. (2022), Zook et al. (2014), Zook et al. (2019) and Zook et al. (2020)
			Quartet	GBW09900/GBW09901/GBW09902/GBW09903	gDNA from B-lymphoblastoid cell lines of a Chinese Quartet family	Yes	Jia et al. (2023), Ren et al. (2023) and Zheng et al. (2023)
		Genetic testing for germline variants	GeT-RM	–	Immortalized cell lines from patients with pathogenic mutations or variants on pharmacogenetic loci	Yes	Amos Wilson et al. (2008), Bettinotti et al. (2018), Gaedigk et al. (2019), Kalman et al. (2007), Kalman et al. (2011), Pratt et al. (2009) and Pratt et al. (2016)
		Whole-genome somatic variants	MAQC/SEQC	HCC1395/HCC1395BL	gDNA from a triple-negative breast cancer and its paired B-lymphoblastoid cell lines	Yes	Fang et al. (2021)
		Whole-exome germline and somatic variants	MAQC/SEQC	SampleA/SampleB	gDNA from ten cancer cell lines and a normal male cell line	Yes	Jones et al. (2021)
		Tumor mutation burden	FCR	–	gDNA from 36 tumor-normal matched FFPE samples	No	Merino et al. (2020), Stenzinger et al. (2019) and Vega et al. (2021)
	Engineered DNA	Cancer associated variants on genomic DNA	MDIC	–	Cancer related variants engineered into DNA sequence of HG002 by CRISPR/Cas9	No	
			SPOT/Dx	–		No	Pfeifer et al. (2022)
			NCCL	–		No	Wang et al. (2023)
		Cancer associated variants on ctDNA	Thermo Fisher	AcroMetrix	Engineered cancer actionable mutations mixed with genomic DNA background offered in series dilution of VAF	Yes	Thermo Scientific (2020)
			SeraCare	Seraseq		Yes	Seracare (2023a, b)b
			Horizon	Mutiplex		Yes	Horizon Discovery (2023)
	Synthetic DNA	Germline and somatic variants	GIMR	sequins	Synthetic mirrored human DNA sequences	Yes	Deveson et al. (2016, 2019)
RNA	Biological RNA	Whole transcriptome	MAQC/SEQC	SampleA/SampleB	Total RNA mixture from ten tumor cell lines and a human brain reference RNA from 23 donors	Yes	MAQC Consortium (2006, 2014)
			Quartet	GBW09904/GBW09905/GBW09906/GBW09907	RNA from B-lymphocyte cell lines of a Chinese Quartet family	Yes	Yu et al. (2023)
		Single-cell RNA-seq	MAQC/SEQC	HCC1395/HCC1395BL	Single-cell gene expression from a triple-negative breast cancer and its paired B-lymphoblastoid cell lines	Yes	Chen et al. (2021)
	Synthetic RNA	RNA	ERCC^1^	ERCC spike-in Mix	92 artificial RNA transcripts	Yes	Jiang et al. (2011) and Munro et al. (2014)
			GIMR	Sequins	Synthetic RNA isoforms with exons and introns	Yes	Hardwick et al. (2016)
		miRNA	ERCC^2^	–	Synthetic RNA oligonucleotides	No	Giraldez et al. (2018)
Protein	Biological protein	Proteins from tissue	NIST	RM8462	Frozen human liver suite consists of normal, fatty and congested liver samples	Yes	Lippa et al. (2022)
		Protein from yeast	CPTAC	–	Digested Yeast Lysate	Yes	Beasley-Green et al. (2012) and Paulovich et al. (2010)
		Protein from cell line	Quartet	Quartet Protein	Peptides digested from B-lymphoblastoid cell lines of a Chinese Quartet family + 4 peptides spike-in	Yes	Tian et al. (2023)
			CPTAC	NCI-7	Proteins from a panel of seven cancer cell lines (NCI-H23, RPMI-8226, T47D, A549, COLO205, NCI-H226, CCRF-CEM)	No	Clark et al. (2018)
			Thermo Fisher	HeLa	Proteins digested from HeLa cell lines	Yes	Kocher et al. (2011)
		Proteins from plasma	NIST	RM8231	Frozen human plasma suite consists of diabetic, high triglyceride, young African-American and Normal plasma samples	Yes	Lippa et al. (2022)
	Synthetic protein	Purified human proteins	Sigma-Aldrich/ABRF	UPS1/2	48 human purified recombinant proteins	Yes	Andrews et al. (2006)
			Invitrogen/HUPO	–	20 human purified recombinant proteins	Yes	Bell et al. (2009)
			NIST/CPTAC	NCI-20	20 human purified recombinant proteins	Yes	Tabb et al. (2010) and Wang et al. (2014)
			Biognosys	iRT	13 peptides	Yes	Escher et al. (2012)
			ABRF/JPT	SpikeMix	1000 peptides	Yes	JPT Peptide Technologies (2023)
Metabolite	Biological metabolite	Metabolites from tissue	NIST	RM 8462	Frozen human liver suite consists of normal, fatty and congested liver samples	Yes	Lippa et al. (2022)
		Metabolites from cell line	Quartet	Quartet Metabolite	Metabolites from B-lymphoblastoid cell lines of a Chinese Quartet family	Yes	Zhang et al. (2022)
		Metabolites from plasma	NIST	SRM 1950	Human plasma of 100 healthy individuals (50 males + 50 females)	Yes	Phinney et al. (2013)
				RM 8231	Human plasma of diabetic, hypertriglyceridemic, African-American and normal (SRM 1950) samples	Yes	Aristizabal-Henao et al. (2020)
				SRM 1949	Human prenatal serum suite consists of non-pregnant and three pregnant trimesters samples	Yes	Boggs et al. (2021) and Sempos et al. (2022)
		Metabolites from urine		RM 8232	Human urine of female non-smokers, female smokers, male non-smokers and male smokers	Yes	Lippa et al. (2022)
	Synthetic metabolite	Synthetic metabolites	CIL	QReSS	18 isotope-labeled metabolite mixes	Yes	Cambridge Isotope Laboratories, Inc. (2023)
			IROA	IROA-LTRS	Yeast extract isotope-labeld complex with universally labeled at both 5% and 95% U-13C and mixed 1:1	Yes	Evans et al. (2020)
			SCIEX	Lipidyzer	Unlabeled lipid mole cular species	Yes	Lippa et al. (2022)
			Biocrates	MxP/AbsoluteIDQ	Seven levels of calibration standards and isotope-labeled internal standards, and plasma-based quality control samples at three concentrations	Yes	Biocrates (2023)

This list provides examples of commonly used representative omics reference materials, rather than an exhaustive compilation of all exiting omics reference materials

CPTAC, Clinical Proteomics Technology Assessment for Cancer; ERCC^1^, External RNA Controls Consortium; ERCC^2^, Extracellular RNA Controls Consortium; FCR, Friends of Cancer Research; FNIH, Foundation for the National Institutes of Health; GIAB, Genome in a Bottle; GeT-RM, Genetic Testing Reference Material Coordination Program; GIMR, Garvan Institute of Medical Research; IFCC, International Federation for Clinical Chemistry; MAQC/SEQC, MicroArray/Sequencing Quality Control; MDIC, Medical Device Innovation Consortium; NIST, National Institute of Standards and Technology; SPOT/Dx, Sustainable Predictive Oncology Therapeutics and Diagnostics

## DNA Reference Materials

DNA RMs are designed to assess the accuracy of genetic variant detection using high-throughput DNA sequencing technologies (Fang et al. 2021; Zook et al. 2014). These materials are available in various formats to suit different research purposes. Biological DNA RMs are typically genomic DNA (gDNA) obtained from natural biological materials, such as Epstein–Barr virus (EBV)-immortalized lymphoblastoid cell lines or tumor cell lines. Immortalized cell lines are convenient and cost-effective sources of reference materials, as they can be readily proliferated through cell culturing, providing a renewable source of gDNA (Fang et al. 2021; Ren et al. 2023; Zook et al. 2014). To ensure that RMs are sufficiently homogenous and in a large quantity to be widely disseminated, they are usually extracted from a single large batch of cell culture and well-mixed. Although subtle genetic differences may exist among cells cultured in different dishes, each vial of reference materials contains the same mixture of genomes, because the cells and gDNA are thoroughly mixed. Biological DNA RMs represent the full size and complexity of the human genome, making them ideal for benchmarking thousands or even millions of variants detected through whole-genome (WGS) and whole-exome sequencing (WES). For genetic testing targeting specific disease-causing variants, patient genomes containing these variants can be provided as valuable reference materials (Kalman et al. 2007; Li et al. 2020). Alternatively, engineered DNA RMs with specific variants introduced into the genome using genome-editing technologies can be used (Lin et al. 2022; Suzuki et al. 2020). When assessing the performance of experimental and bioinformatics processes, DNA RMs derived from natural or engineered cell lines are typically analyzed in parallel alongside study samples, whereas synthetic spike-in controls are usually added to samples of interest as internal controls throughout the entire sequencing workflow to measure technical artifacts. To distinguish them from study samples, synthetic spike-in controls are composed of non-human artificial DNA sequences or contain unique molecular barcodes (Blackburn et al. 2019; Reis et al. 2020).

### Biological DNA Reference Materials

Germline variants are inherited genetic changes that occur in either a sperm or an egg cell and are passed on to offspring at the time of conception. These variants are present in all cells of the body and are typically detected from a blood sample. The accurate and reliable detection of germline variants is crucial for identifying genetic causes of disease and developing personalized treatment strategies.

The GIAB consortium, hosted by NIST, is dedicated to creating reference materials, methods, and datasets that facilitate the clinical translation and regulation of human genome sequencing (National Institute of Standards and Technology 2023b). In 2015, NIST released the primary human genome DNA reference material, RM 8398, derived from HG001/NA12878, a healthy female of European ancestry. To improve the representation of human genetic diversity, NIST further developed DNA reference materials from different ethnic populations, including an Ashkenazi Jewish family trio (RM8392) and a Han Chinese son (RM8393) (Zook et al. 2016). These genomes were chosen from the Personal Genome Project because of the broad consent for public genome data sharing and commercial use of products based on these cell lines. This broad consent has enabled commercial reference materials to be based on the same cell lines characterized by the GIAB, including spike-in DNA mimicking challenging variants, somatic variants, and circulating tumor DNA (ctDNA), which are explained below. The seven genomes have been extensively characterized by the GIAB consortium for benchmarking germline variants, including single nucleotide variations (SNVs), small indels and structural variants (SVs) (Wagner et al. 2022; Zook et al. 2014, 2019, 2020). While all of GIAB's current benchmarks are focused on germline "normal" cell lines, the consortium is currently collaborating to develop new broadly consented tumor-normal cell line pairs for genomic RM development.

In recent years, several initiatives have made significant strides in developing biological DNA RMs to serve as benchmarks for whole-genome germline variants (Li et al. 2018). The Quartet Project, led by Fudan University in close collaboration with the National Institute of Metrology of China and other organizations, established four immortalized lymphoblastoid cell lines from a Chinese Quartet family, including a father, mother and two monozygotic daughters (Ren et al. 2023; Zhang et al. 2023). This family was recruited from the Fudan Taizhou cohort in Central China, thus possessing genetic features from both Northern and Southern Chinese populations (Wang et al. 2009). The four DNA RMs have been certified by China's State Administration for Market Regulation as the First Class of National Reference Materials. They have been extensively used for proficiency testing and methods validation in clinical and commercial laboratories and the sequencing datasets are publicly available (Khayat et al. 2021; Pan et al. 2022). In addition to DNA RMs, the Quartet Project released corresponding RNA, protein and metabolite RMs derived from the same cell lines. Therefore, the Quartet RMs have three types of “truth” to assess the performance of variants calling results (Ren et al. 2023). The first is the characterized benchmark variants, which can be used to evaluate the performance of variants identified inside the benchmark regions. The second is the Mendelian inheritance law underlying the monozygotic twins and their parents. The third kind of “truth” is central dogma of multiomics RMs, which enables cross-omics validation of variant calls from multiomics datasets.

High-throughput sequencing technologies aim to scan variants on a whole-genome scale, while clinical genetic testing focuses on a few particular genetic variants associated with diseases. To support quality control for clinical genetic testing, the U.S. Centers for Disease Control and Prevention (CDC) led the Genetic Testing Reference Materials Coordination Program (GeT-RM) to develop DNA RMs, including those for rare inherited genetic diseases, human leukocyte antigen (HLA) testing and pharmacogenetics (Coriell Institute 2023). The GeT-RM obtained cell lines containing medically important mutations from the Coriell Cell Repositories, and then distributed genomic DNAs to multiple volunteering laboratories for genotyping and mutation confirmation using a variety of platforms and assays (Centers for Disease Control and Prevention 2019). The GeT-RM has characterized DNA RMs for a wide range of genetic disorders, such as cystic fibrosis (Pratt et al. 2009), Duchenne and Becker muscular dystrophy (Kalman et al. 2011), fragile X syndrome (Amos Wilson et al. 2008), Huntington disease (Kalman et al. 2007), and many others, including 11 human leukocyte antigen loci (Bettinotti et al. 2018) and pharmacogenetic loci (Gaedigk et al. 2019; Pratt et al. 2016). These reference materials represent specific mutations associated with diseases and are available for research, clinical test development, quality assurance and control, and proficiency testing to ensure the accuracy of clinical testing.

Somatic variants are genetic mutations that occur in non-germline cells. They are typically detected in tumors from sequencing datasets of paired tumor and normal samples, with normal samples used to remove germline variants. Accurate and reliable detection of somatic variants is crucial for gaining insights into cancer biology, guiding targeted therapies and improving patient outcomes in cancer treatment. DNA RMs used to benchmark somatic variants usually consist of matched tumor and normal genomes.

The MicroArray and Sequencing Quality Control (MAQC-IV/SEQC2) consortium recently completed its fourth project, which aimed to develop standard analysis protocols and quality control metrics for the use of high-throughput DNA sequencing data in regulatory science research and precision medicine (MAQC Consortium 2021). The Somatic Mutation Working Group (WG1) of SEQC2 established paired tumor-normal DNA RMs and corresponding whole-genome reference datasets for small variants and structural variants (Fang et al. 2021; Talsania et al. 2022). The DNA RMs are gDNA derived from a triple-negative breast cancer (TNBC) cell line (HCC1395) and a B-lymphocyte-derived normal cell line (HCC1395BL) from the same donor, obtained from the American Type Culture Collection (ATCC). HCC1395 is a well-studied cell line with abundant somatic alterations, including approximately 40,000 SNVs, around 2000 indels, copy number alterations (CNAs) affecting 56% of the genome, 256 complex genomic rearrangements, and 138 experimentally confirmed fusion genes (Stephens et al. 2009). The SEQC2 WG1 later used DNA RMs to address challenges in accurately detecting somatic variants from WGS and WES by examining experimental and bioinformatic components affecting their reproducibility and accuracy, covering a wide range of topics, including library preparation protocols, DNA input amount, tumor purity, read coverage, and bioinformatic pipelines (Sahraeian et al. 2022; Talsania et al. 2022; Xiao et al. 2021).

Due to the heterogeneity of tumors and diverse mutational profiles among different types of cancer, a single reference material, such as HCC1395, may not fully represent breast or any other types of cancer genomes. Nevertheless, it is highly mutated and suitable for benchmarking, developing, and refining protocols and tools for somatic variant detection. To better capture the genetic diversity of tumor genomes, researchers have made efforts to establish DNA RMs from more tumor types. For instance, Craig et al. (2016) created DNA RMs from a metastatic melanoma (COLO829) and its paired B-lymphoblastoid normal cell line (COLO829BL).

While WGS and WES provide a more comprehensive view of the entire genome, targeted sequencing, also known as oncopanel sequencing, offers a more cost-effective and efficient approach by focusing on a limited number of cancer hotspot variants. It can detect variants with a variant allele frequency (VAF) as low as 0.5%. The Oncopanel Sequencing Working Group (WG2) of SEQC2 established two DNA RMs for oncopanel benchmarking (Jones et al. 2021). Sample A is an equal mass pooled gDNA sample of the same 10 cancer cell lines that were originally used for developing the Agilent Universal Human Reference RNA material (UHRR, Catalog #74000) (MAQC Consortium 2006, 2014), covering as many clinically related variants as possible to increase variant density in coding regions. Sample B is derived from a non-cancer male cell line (Agilent OneSeq Human Reference DNA, PN 5190-8848). To emulate the range of VAFs typically encountered in targeted sequencing and ctDNA sequencing, tumor Sample A was diluted by normal Sample B at different ratios to create a series of tumor DNA reference materials with even lower VAFs of variants. The SEQC2 WG2 employed these DNA RMs to conduct cross-platform multi-laboratory evaluations of commercially available oncopanels, and developed actionable guidelines to improve the performance and consistency of oncopanel sequencing across different laboratories and platforms (Deveson et al. 2021a; Gong et al. 2021).

Apart from benchmarking individual somatic variant calls, DNA RMs have been developed to benchmark aggregated genomic biomarkers derived from somatic variants, such as tumor mutation burden (TMB). TMB is a promising biomarker for predicting response to pan-cancer immune checkpoint inhibitor therapy (Samstein et al. 2019; Yarchoan et al. 2017). The gold standard for measuring TMB is to perform tumor-normal paired WES and count the total number of non-synonymous mutations in the coding regions. However, WES is a relatively costly and time-consuming approach. To address this, researchers are exploring the use of less expensive targeted sequencing panels that focus on a small number of driver genes to estimate TMB. However, significant variability in TMB measurement has been observed (Buttner et al. 2019). To address the need to standardize and harmonize TMB assessment across assays and laboratories, many initiatives have developed DNA RMs, such as Friends of Cancer Research TMB Harmonization Project (Merino et al. 2020; Stenzinger et al. 2019; Vega et al. 2021) and SeraSeq (Seracare 2023a). These DNA RMs are established from Formalin-Fixed Paraffin-Embedded (FFPE) clinical samples or tumor cell lines. Contrived RMs are developed by mixing gDNA of tumor cell lines with matched normal cell lines at a series of proportions to mimic low VAF variants detected from liquid biopsy (Zhang et al. 2021).

### Engineered DNA Reference Materials

Engineered DNA RMs are designed to assess the analytical performance of laboratory developed tests (LDTs) for oncology therapies by introducing desired cancer hotspot mutations into germline DNA RMs using gene editing systems such as CRISPR/Cas9 (Jia et al. 2018; Pfeifer et al. 2022; Suzuki et al. 2020; Zehnbauer et al. 2017). Cell lines like HapMap cell lines (e.g., HG001 and HG002) and the Quartet cell lines are preferred for this purpose, because they can be easily cultured in large quantities and widely distributed due to their broad consent (Lin et al. 2022). Each variant is independently engineered into different cell lines, and the multiplexed DNA RMs are created by mixing genomes with engineered variants together (Medical Device Innovation Consortium 2019). The risk of unexpected off-target effects induced by genome editing is a major concern, which can result in variants at similar DNA sequences other than the intended on-target sites (Zhang et al. 2015). To ensure that the original and engineered cell lines are isogenic at all locations except for the engineered variant sites, various computational and experimental methods are used to detect any off-target CRISPR/Cas9 activity, including Sanger sequencing, Food and Drug Administration (FDA)-approved clinically validated targeted gene panels, PacBio WGS, and circularization for high-throughput analysis of nuclease genome-wide effects by sequencing (CHANGE-seq) technology (Lazzarotto et al. 2020). Engineered DNA RMs can also be developed with abundant somatic variants by knocking down genes in the mismatch repair (MMR) pathway and proofreading systems, which are crucial for high fidelity of genome replication (Wang et al. 2023). Clones are then selected by flow cytometry and cultured to accumulate sufficient somatic variations. One major concern of engineered DNA RMs is that the engineered genomes are not able to mimic the complexity and heterogeneity of real cancer genomes.

Engineered DNA RMs can be used to benchmarking ctDNA assays (Deveson et al. 2021b; Horizon Discovery 2023; Seracare 2023b; Thermo Scientific 2020). To emulate low concentrations of ctDNA in plasma, the mutated genome is diluted by a background genome with wild-type alleles, resulting in somatic variants with low VAF. The DNA sequences are then fragmented to an average size of 150–170 bp to closely resemble highly degraded ctDNA extracted from human plasma. While researchers have attempted to digest DNA sequences using micrococcal nuclease (MNase) to preserve nucleosome core particles and trimmed nucleosomes (Zhang et al. 2017), further investigation is needed to determine if the contrived DNA RMs can fully represent the biological properties of ctDNA and perform equivalently, or even sufficiently, to clinical specimens.

### Synthetic DNA Reference Materials

Synthetic DNA molecules are artificially created through chemical synthesis techniques and do not necessarily align with the human reference genome. The synthetic DNA RMs are developed to represent diagnostic features and to address the specific requirements of any next-generation sequencing (NGS) test, especially clinically relevant or difficult variants. Synthetic DNA RMs are often used as spike-in controls to be added into a study sample with a known quantity to measure sensitivity and precision of NGS libraries (Deveson et al. 2016, 2019). These spike-ins should be added at sufficient abundance to achieve matched sequencing coverage with the accompanying sample without sacrificing too many sequencing reads (Blackburn et al. 2019).

Synthetic DNA RMs enable researchers to evaluate the quantitative properties of DNA sequencing, such as VAF, limit of detect (LOD), and copy numbers. A pair of synthetic DNA RMs, which represent wile-type and mutant alleles, can be combined to simulate lower somatic VAF and to establish LOD (Blackburn et al. 2019; Deveson et al. 2021b). Alternatively, sequence elements can be encoded in a single synthetic DNA molecule at known abundances (Reis et al. 2020).

## RNA Reference Materials

RNA sequencing (RNA-seq) is typically used for identifying differentially expressed transcripts or genes between experimental groups and control groups; thus, RNA RMs are often provided as sample pairs or groups. As high-throughput sequencing technologies do not directly measure absolute abundances of RNA molecules reliably, differences or relative expression levels (ratios) between sample groups serve as a built-in truth to benchmark quantitative measurements, instead of absolute abundance for each RNA in a single sample. Biological RNA RMs are derived from single large batches of immortalized cell lines. Gene and transcript expression levels are characterized by multiple methods (MAQC Consortium 2006; Yu et al. 2023). Synthetic RNA RMs are exogenous or artificial RNA oligonucleotides with a wide range of known concentrations, which enable for the assessment of dynamic range, specificity, sensitivity and LOD that is not otherwise possible for biological RNA reference materials (Jiang et al. 2011; Munro et al. 2014). However, synthetic RNA RMs lack the desired complexity and diversity and, thus, could behave differently from biological samples.

### Biological RNA Reference Materials

The MAQC/SEQC projects initiated by the US FDA utilized two human RNA RMs to comprehensively evaluate the comparability and accuracy of gene-expression measurements obtained through microarray and RNA-seq techniques across different laboratories and protocols (MAQC Consortium 2006, 2014). The two RNA RMs are the Agilent Universal Human Reference RNA composed of total RNA from 10 human tumor cell lines (termed “Sample A”) and the ThermoFisher Human Brain Reference RNA (HBRR, termed “Sample B”). A pair of RNA RMs can be combined together at known mixing ratios, thus enabling users to assess relative accuracy of each method based on the differentially expressed genes detected. Samples A and B were then mixed in 3:1 and 1:3 ratios, respectively, to generate Samples C and D. This combination of biologically different RNA sources and known titration differences enables assessment of relative accuracy based on the differentially expressed genes detected.

In addition to comparing the consistency of differentially expressed genes between query datasets and reference datasets, unsupervised clustering methods such as principal component analysis (PCA) were used to assess the performance of omics data to distinguish sample groups. Gene expression profiles between MAQC samples A and B are significantly different with more than 15,000 differentially expressed genes. Successfully distinguishing sample groups with such large differences does not guarantee the ability to identify subgroups with subtle biological differences of clinical samples. The four Quartet RNA reference materials are much more similar to each other and require increased performance of a method for distinguishing them (Yu et al. 2023). There are about 2000 differentially expressed genes between any two of the Quartet samples, which are enriched in B cell mediated immunity. Triplicates of each Quartet RNA reference material were sequenced in a batch to benchmark transcriptomic profiling cross protocols and laboratories. The signal-to-noise ratio (SNR) metric, defined as the ratio of the inter-sample distance on the PCA plot over the intra-sample distance between technical replicates, is applied to assess the quality of query datasets. Higher SNR values indicate stronger power to discriminate sample groups, while lower SNR values indicate technical biases or sequencing failures of one or more replicates. In the Quartet benchmark studies, some experiments were found to have high replicate consistency but relatively low SNR values, indicating that these experiments may had systematic technical biases which can only be revealed by multi-sample reference materials.

### Synthetic RNA Reference Materials

Synthetic RNA RMs are used as external spike-in controls to be added to the samples of interest for transcriptomic analysis. Laboratories have used different custom external spike-ins for specific platforms and assays, before the External RNA Controls Consortium developed the first generally accepted RNA spike-ins for various microarray and sequencing applications, which were later distributed by NIST as SRM 2374 (Baker et al. 2005). The ERCC spike-in controls comprise 92 polyadenylated RNA transcripts derived from bacterial sequences or in vitro transcription of synthetic DNA sequences. The 92 ERCC RNA control transcripts are categorized into four sub-pools, each containing 23 transcripts spanning a wide dynamic range in concentration. The sub-pools are combined to create two mixtures, Mix1 and Mix2, in four defined abundance ratios of 4:1, 1:2, 1:1.5, and 1:1 (Munro et al. 2014). Spike-in controls can be added to biological RNA RMs to combine the advantages of both types of reference materials into a single sequencing run. In the third phase of the MAQC project or SEQC, researchers conducted a broader analysis of RNA-seq performance evaluating the sensitivity and technical variation between different NGS methods and laboratories by complementing samples A and B with ERCC controls (MAQC Consortium 2014).

While ERCC spike-in transcripts are valuable for standardization and quantification in transcriptomic analysis, they have limitations in representing the diversity and complexity of endogenously expressed transcripts due to their lack of isoforms. To address this limitation, alternative RNA reference materials have been developed. Sequins, designed by the Garvan Institute of Medical Research, provide a comprehensive representation of alternative isoforms and the complex exon–intron architecture of human genes, allowing for more accurate assessment of gene fusion, alternative splicing, and transcript assembly (Hardwick et al. 2016). Additionally, the spike-in RNA variants (SIRV) developed by Lexogen consist of 69 synthetic transcript isoforms that comprehensively reflect variations of alternative splicing, alternative transcription start- and end-sites, overlapping genes, and antisense transcripts (Lexogen 2023). This set of RNA reference materials enables the evaluation of the performance of isoform-specific RNA-seq workflows, and thus provides a more comprehensive evaluation of RNA-seq performance.

RNA-seq can be used to sequence long RNAs, such as messenger RNAs, as well as short RNAs, such as microRNAs (miRNAs), that differ in length. The Extracellular RNA Communication Consortium led a benchmark study for miRNA quantification across multiple protocols and laboratories using small RNA-seq (Giraldez et al. 2018). They used diverse combinations of synthetic RNAs to evaluate sequence-specific biases and accuracy. An equimolar pool consisted of over 1000 chemically synthesized RNA oligonucleotides (15–90 nt) mixed at equal concentration was used to assess reproducibility of absolute RNA sequences abundance at counts per million (CPM) level. Two synthetic small RNA pools with RNAs varied in defined relative amount were used to assess the concordance for relative quantification. Synthetic pools with unedited and edited miRNA variants in different ratios were used to determine the accuracy of quantifying miRNA editing.

## Protein Reference Materials

The proteome refers to the entire set of proteins expressed by a cell, tissue or organism at a particular time. Proteomics is the systematic, high-throughput study of the composition, functions, and interactions of all proteins. In proteomics research, where the sheer multitude of proteins presents a formidable challenge, mass spectrometry (MS) is commonly used for both qualitative and quantitative protein analysis. This process involves comparing detected peptide maps with protein sequences sourced from databases. However, the complexity of MS-based proteomics experiments and their potential for considerable variability can hinder the achievement of accurate and reproducible results. To enhance the reliability and reproducibility of proteomics, numerous initiatives have been actively working for decades to establish community standards and guidelines. These efforts aim to ensure consistency, promote rigorous experimental practices, and facilitate the generation of reliable and comparable proteomic data across different laboratories and studies. For example, the Proteomics Standards Initiative (PSI) of the Human Proteome Organization (HUPO) standardized practices and guidelines for data reporting formats (Deutsch et al. 2017), data quality control framework (Bittremieux et al. 2017) and data interpretation (Omenn 2021). CPTAC, launched by the US National Cancer Institute (NCI), intends to improve MS-based proteomics measurement quality for biomarker discovery in cancer research (Tabb et al. 2016; Zhou et al. 2017). The Proteomics Standards Research Group (sPRG) of the Association of Biomolecular Resource Facilities (ABRF) develops and implements standards to reflect the accuracy and consistency of proteomics (Tabb et al. 2010).

The limitations of traditional methods have driven the development of new technologies tailored for highly sensitive protein biomarker discovery while demanding minimal quantities of biological materials (Eldjarn et al. 2023; Sun et al. 2023). Examples of this innovation include Olink's Proximity Extension Assay (PEA) (Petrera et al. 2021; Wik et al. 2021), SomaLogic's SomaScan Assay (Candia et al. 2017, 2022), and Seer's Proteograph (Blume et al. 2020). Among them, the Olink technology implements more stringent quality control procedures by integrating four internal controls into all samples and including external controls within each plate (Olink 2023). The Olink technology employs a detection principle where two specific proximity probes are coupled, generating an amplicon upon binding to the target protein, which is then quantified using quantitative real-time PCR (qPCR) or NGS (Wik et al. 2021). The internal controls encompass an incubation control, an extension control, and a detection control, each serving distinct roles in data quality assurance during the PEA. The incubation controls involve non-human antigens with matching antibodies for monitoring throughput the PEA process. The extension control consists of IgG antibodies coupled with matching oligo pairs, ensuring the constant proximity of DNA tags and supporting data normalization while monitoring the extension, amplification, and detection steps. The detection control, a synthetic double-stranded DNA, contributes to data quality control and aids in identifying potential issues in the final amplification and detection stages. Furthermore, each sample plate incorporates eight external controls, which consist of two pooled sample controls to estimate inter and intra consistency for each assay, three negative controls containing buffer to establish background levels and calculate the limit of detection, and three plate controls designed to account for potential variations between runs and plates, further enhancing the reliability and accuracy of the results.

In the realm of proteomics studies, the inclusion of various types of RMs, such as internal standards, blank QC samples or negative controls, and pooled QC samples, equips researchers with the means to effectively manage variability, achieve precise quantification, and ensure the reliability of their findings (Bittremieux et al. 2018; Bunk 2010; Chiva et al. 2021). Within this review, our primary focus is on external RMs that can be analyzed alongside the study samples, enabling comparisons between runs, batches and studies, as well as ensuring the accuracy of detection through comparison with reference datasets or the categorization of different sample groups.

A diverse range of protein RMs has been developed to support both qualitative and quantitative proteomic measurements, serving as invaluable tools for method validation, quality control, and calibration within the field of proteomics research. These RMs serve as valuable tools for method validation, quality control, and calibration in proteomics research. These RMs vary in complexity, ranging from simple synthetic peptide mixtures to digests of bovine serum albumin and even complex human tissues (Bittremieux et al. 2018). Biological matrix protein RMs are derived from biological materials such as whole-cell lysate and bio-fluids, offering a higher level of sample complexity with tens of thousands of proteins. While biological protein RMs obtained from human tissues and plasma better resemble the biological features of their respective sample types, their availability is usually limited, and the reference datasets can differ between lots. In contrast, microorganisms and cell lines provide inexpensive and renewable protein RMs, ensuring sufficient quantities for the research community. Synthetic protein RMs, on the other hand, consist of mixtures of a limited number of purified recombinant human proteins or chemically synthesized peptides with defined molecular weights and concentrations. However, the composition of synthetic proteins is much simpler and does not resemble the complex biological characteristics of clinical samples.

### Biological Protein Reference Materials

Protein identification is a critical component of proteomics and often leads to subsequent investigations in which these proteins are quantified. One-sample based RMs are often used to evaluate the performance of protein identification and absolute quantification, for example RM8461 released by NIST from a cryogenically homogenized and freeze-dried liver tissue (Davis et al. 2019), yeast *Saccharomyces cerevisiae* (later released by NIST as RM8323) (Beasley-Green et al. 2012; Paulovich et al. 2010), bacterium *Shewanella oneidensis* (Nakayasu et al. 2021), NCI-7 Cell Line Panel (Clark et al. 2018), HeLa cells (Kocher et al. 2011) and HEK293T cells (Collins et al. 2017). While these proteomes have been extensively characterized and served as a model proteome in numerous fundamental proteomic investigations, reference datasets of high-confidence proteins have not yet been established. As a result, the performance of protein identification and absolute quantification are currently being assessed primarily based on the consistency between technical replicates of the same RM.

Another important application of quantitative proteomic measurements is to determine differentially expressed proteins (Anwaier et al. 2022; Ku et al. 2023). Multi-sample RM suites are developed to facilitate relative quantitation assessment. CPTAC established a pair of patient-derived xenograft tumors as a comparative reference material (CompRef) to longitudinally monitor the reproducibility of differential proteomics across instruments and centers for the Cancer Genome Atlas (TCGA) (Tabb et al. 2016; Zhou et al. 2017). The CompRef represent basal (WHIM2) and luminal-B (WHIM16) breast cancer subtypes, having significantly different proteomic signatures. They were then utilized as standards to establish uniform analytical pipelines for other cancer types (colorectal and ovarian cancers) by the CPTAC Common Data Analysis Platform (CDAP) (Rudnick et al. 2016). NIST is currently developing protein RMs suites for the assessment of relative quantitation in proteomics, including RM 8462 Frozen Human Liver Suite (normal, fatty and congested liver samples) and RM 8231 Frozen Human Plasma Suite (Diabetic, high triglyceride, young African-American and Normal plasma samples). The Quartet Project also released biological protein RMs, which are extracted from four lymphoblastoid cell lines as the same with DNA and RNA reference materials mentioned above (Tian et al. 2023).

### Synthetic Protein Reference Materials

Synthetic protein reference materials have been extensively used in benchmark studies of proteomic measurements to determine experimental and analytical variations by big consortia (Paulovich et al. 2010; Tabb et al. 2010). Notable examples of standard protein mixtures include: the Universal Proteomics Standards (UPS1 and UPS2), a mixture of 48 human recombinant proteins jointly developed by ABRF's sPRG and Sigma-Aldrich (Andrews et al. 2006); the HUPO Gold MS Protein Standard, a mixture of 20 human proteins, developed by the joint efforts of HUPO and Invitrogen (Bell et al. 2009); a mixture of 20 purified human proteins (NCI-20), produced by NIST and employed by CPTAC for intra- and inter-laboratory studies aiming at evaluating repeatability and comparability of qualitative proteomics (Tabb et al. 2010; Wang et al. 2014).

Chemical synthetic or modified peptide mixtures are also utilized as RMs. In comparison to protein mixtures, peptide mixtures have a simpler composition. However, it is important to note that they cannot fully capture the variability introduced during enzymatic digestion, as different laboratories may employ diverse proteolytic enzymes, chemicals, and conditions for digestion. Several synthetic peptide reference materials are commercially available, such as a mixture of 1000 heavy-label proteotypic peptides for conserved proteins across three species (human, mouse and rat), established by ABRF and JPT Peptide Technologies (2023). Synthetic peptides are especially important to evaluate the performance of targeted quantitative proteomic measurement, such as multiple reaction monitoring (MRM) and parallel reaction monitoring (PRM). They are often used to predict retention times (RTs) for large-scale scheduled liquid chromatography multiple reaction monitoring (LC-MRM) measurements with a single calibration run before the analytical runs. Biognosys has developed a mixture of 11 artificial synthetic peptides (iRT) to determine peptide retention time (RT) values and calibrate chromatographic systems for increasing the throughput (Escher et al. 2012). Additionally, well-defined synthetic protein reference materials can be added into biological protein reference materials or test samples to provide additional information of qualitative accuracy. An important consideration when spiking synthetic peptides into other samples is that these peptides should not overlap with the original sample content.

## Metabolite Reference Materials

Metabolomics encompasses the extensive investigation of small molecules, known as metabolites, within cells, biological fluids, tissues, or organisms. It integrates the influences of factors from genomics, transcriptomics, proteomics, as well as environmental elements like diet and lifestyle. Since metabolites serve as indicators of the downstream effects of these factors on cellular functions, they closely represent the actual phenotypes of cells, tissues, or organisms, offering novel insights into metabolism and its regulation in physiological and pathological processes, including health, aging, and diseases. Metabolomics involves the simultaneous identification and quantification of various small molecule types, including amino acids, fatty acids, carbohydrates, and other products of cellular metabolic functions. In comparison to genomics, transcriptomics, and proteomics, the reliable identification and quantification of the metabolome are significantly more complex due to the chemical complexity and the presence of isomers—compounds with the same molecular formula but different structural arrangements—introducing challenges for precise identification and quantification.

To promote the advancement of metabolomics toward higher quality, several large research consortia have emerged in the field, aiming to enhance the reproducibility of metabolomics research results through comprehensive quality assurance and quality control measures. These consortia have undertaken various efforts, including the establishment of best practices, promotion of communication and education, and the advancement of the field toward higher-quality standards. The mQACC, consisting of experts in quality assurance and quality control, is focused on developing universal best practices and reporting standards to ensure the robustness and reproducibility of untargeted metabolomics research (Beger et al. 2019; Evans et al. 2020). The Metabolomics Society Data Quality Task Group (DQTG) aims to enhance the robustness of quality assurance and quality control in the metabolomics community through communication, advocacy, education, and the promotion of best practices (Kirwan et al. 2022). The Standard Metabolic Reporting Structures (SMRS) group is dedicated to standardizing metabolomics analysis and provides comprehensive reports and summaries on relevant key issues (Beckonert et al. 2007; Lindon et al. 2005). The ABRF Metabolomics Research Group aims to study the reproducibility of metabolomics research and propose best data analysis strategies by comparing analysis groups using the same dataset (Turck et al. 2020). Additionally, the ABRF plays a role in improving the core competencies of biotechnology laboratories through research, communication, and education (Cheema et al. 2015; Turck et al. 2020). The Metabolomics Consortium has proposed guidelines for achieving high-quality reporting of LC–MS-derived metabolomics data, including the identification and prioritization of test materials, assessment of useful indicators of data quality, and descriptions of common practices and variations in quality assurance and quality control workflows (Broadhurst et al. 2018).

Quality control samples can be categorized into three primary types based on their intended purposes. System suitability test samples serve as a quality assurance measure applied before data acquisition to instill confidence in the eventual high-quality results (Broadhurst et al. 2018; Kirwan et al. 2022). Typically, these samples consist of solutions containing a small number of authentic chemical standards, typically ranging from five to 10 analytes, with known concentrations. They play a critical role in instrument calibration and assessment of critical system parameters, including mass-to-charge (m/z) ratio and chromatographic characteristics such as retention time, peak area, and peak shape.

Blank quality control samples and matrix-matched quality control samples are essential components of quality control measures to ensure that the quality management process is fulfilled. Blank quality control samples consist of samples devoid of metabolites, serving to identify potential sample contamination or instrument-related background signals, thereby eliminating interference from external contaminants or instrument-related background signals, thereby eliminating interference from external contaminants or the instrument itself (Kirwan et al. 2022). By comparing data from the actual samples to that from the blank samples, researchers can distinguish genuine metabolite signals from potential interferences or background noise. Within the category of matrix-matched quality control samples, the most commonly used are pooled samples. These samples are created by pooling a small amount of each analyzed biological sample within a study, representing both the sample matrix and metabolite composition. Pooled QC samples play a multifaceted role, conditioning the analytical platform, conducting intra-study reproducibility measurements, and mathematically correcting for systematic changes in parameter values (Broadhurst et al. 2018). A specific type of pooled QC sample can be used to assess data quality across different studies within the same laboratory, termed long-term reference (LTR) QC samples (Broadhurst et al. 2018). These samples are obtained either through the commercial purchase of the required sample types or by collecting representative samples from various studies within the laboratory. In this review, we focus on the use of external RMs for assessing performance across different laboratories, which are created and sold by a certified group.

### Biological Metabolite Reference Materials

SRM 1950 released by NIST is one of the first developed metabolite reference materials, which is intended for quality control of identifying and quantifying metabolites in human plasma, such as fatty acids, electrolytes, vitamins, hormones, and amino acids (Phinney et al. 2013). It is a mixture of human plasma samples from 100 individuals reflecting a racial distribution in the US population at the time of implementation (77% white, 12% African-American or black, 2% American Indian or Askan Native, 4% Asian, 5% other, with about 15% Hispanic origin). A total of 90 metabolites are assigned with high confidence values of absolute concentrations by integrating several different analytical methods. SRM 1950 was initially designed for targeted metabolomics, and has been extensively used to benchmark platforms, protocols and workflows (McGaw et al. 2010; Misra and Olivier 2020; Siskos et al. 2017; Thompson et al. 2019). Recently, it has also been used in benchmark studies of untargeted metabolomics and lipidomics (Azab et al. 2019; Bowden et al. 2017; Cajka et al. 2017). NIST also released other standalone natural-matrix reference materials for organic contaminants from an assortment of biological materials, including frozen non-fortified human milk (SRM 1953), fortified human milk (SRM 1954), non-fortified human serum (SRM 1957), fortified human serum (SRM 1958) (Schantz et al. 2013), lyophilized human serum (SRM 909b and SRM 909c) (Aristizabal-Henao et al. 2021), smokers' human urine (SRM 3672), and non-smokers' urine (SRM 3673).

Like other quantitative omics, such as transcriptomics and proteomics, identifying differentially expressed metabolites between sample groups is one of the main purposes for metabolomics-based biomarker researches. RMs consisting of two or more sample groups can be used to assess the performance of distinguishing sample groups. The NIST Metabolomics Quality Assurance and Quality Control Materials (MetQual) Program released a suite of pooled plasma materials (RM 8231) comprising four different metabolic health states, including type 2 diabetes plasma, hypertriglyceridemia plasma, normal African-American plasma and normal human plasma (SRM 1950) (Met Qual Program Coordinators 2023). The MetQual Program is planning to conduct an inter-laboratory study to obtain consensus characterization of RM 8231 and assess measurement variability within the metabolomics community. NIST also developed several multi-sample metabolite reference materials from other biological resources. RM 971a consists of two serum mixtures: one from a pool of healthy, premenopausal adult females, and the other one from a pool of healthy adult males. It is intended to evaluate the accuracy of identify and quantify hormones in human serum (Aristizabal-Henao et al. 2021). SRM 1949 Frozen Human Prenatal Serum is a four-level material that was pooled from non-pregnant women and women during each trimester of pregnancy, aiming at quality control for the measurement of hormones and nutritional elements throughout pregnancy (Boggs et al. 2021; Sempos et al. 2022). A suite of human urine reference materials (RM 8232) is under development. The suite will consist of four pooled urine samples from female non-smokers, female smokers, male non-smokers and male smokers. Relative metabolite fold changes, percent differences for the top 20 metabolites and the identified top 30 abundant metabolites of the urine samples will be characterized by both LC–MS and nuclear magnetic resonance. RM 8462 Frozen Human Liver Suite mentioned in the protein reference materials section can be also used for metabolomics (Lippa et al. 2022).

The Quartet Project also developed a multi-sample metabolite RM suite by extracting metabolites from the four immortalized lymphoblastoid cell lines. Aiming at assessing the performance of detecting biological differences between different sample groups, reference datasets for fold changes of absolute abundance values between samples groups were constructed, by consensus across platforms, laboratories and replicates. The performance of quantitative metabolomics can be assessed not only by the consistency between fold changes of differentially expressed metabolites in query datasets and reference datasets, but also by SNR by measuring the ability to discriminate the intrinsic biological differences between the four sample groups.

### Synthetic Metabolite Reference Materials

Synthetic metabolite reference materials are artificial substances that have identical chemical properties to naturally occurring metabolites in biological systems. They play an important role as calibration standards for analytical methods to allow accurate identification and quantification of metabolites. Synthetic metabolite RMs contain known concentrations of chemical components, which can be run separately or used as internal standards to perform system suitability tests, calibration, and metabolite quantification. These RMs can be prepared in individual laboratories to fit specific purposes for each study or can be purchased from vendors. They can be produced using chemical synthesis or enzymatic reactions, and they can be used for a range of applications, including targeted and untargeted metabolomics, and in the development and validation of new analytical methods. Synthetic metabolite RMs can also be used to assess the accuracy and precision of different analytical platforms and to facilitate inter-laboratory comparisons.

One example of a synthetic metabolite RM is the deuterated internal standards that are frequently used in MS-based metabolomics. These internal standards are made by incorporating deuterium into the metabolite of interest, allowing for accurate quantification of the metabolite in biological samples. Commercially available synthetic metabolite reference materials are typically mixtures of isotopically labeled or U-13C labeled metabolites that span a broad range of molecular weights, possess varied ionization propensities, and cover a distribution in class and retention time. Examples of commercially available synthetic metabolite reference materials include the QReSS kit from Cambridge Isotope Laboratories (CIL) (Cambridge Isotope Laboratories, Inc. 2023), the IROA-Long-Term Reference Standard (IROA-LTRS) from IROA Technologies (Evans et al. 2020), the Lipidyzer Platform kits from SCIEX (Lippa et al. 2022), and quantitative metabolic profiling kits from Biocrates (Biocrates 2023).

## Multiomics Reference Materials

Multiomics integrates diverse omics data to better cluster and classify sample (sub)groups, and more comprehensively understand the mechanisms underlying biological processes by investigating molecular interaction across omics layers (Karczewski and Snyder 2018; Price et al. 2017; Schussler-Fiorenza Rose et al. 2019). Multiomics analysis inherits challenges from the single omics datasets and confronts new challenges in data harmonization and integration across different omics layers with varying numbers of features and statistical properties (Athieniti and Spyrou 2023; Sonia Tarazona 2021). Multiomics RMs derived from the same source that incorporate multiple omics types and provide unbiased ground truth serve as crucial tools for assessing the performance of methods for normalizing and integrating multiomics datasets, conducting cross-omics validation, and imputing missing data (Krassowski et al. 2020; Zheng et al. 2023). They enable the validation and comparison of data integration methods across multiple omics layers, allowing for the identification of potential biases or discrepancies in the integration of data. Cross-omics validation is a critical step in multiomics research, involving comparing and validating findings across different omics layers. Multiomics RMs provide a standardized framework for conducting such validation, ensuring that results obtained from different omics techniques align and mutually reinforce each other.

**Fig. 1:**
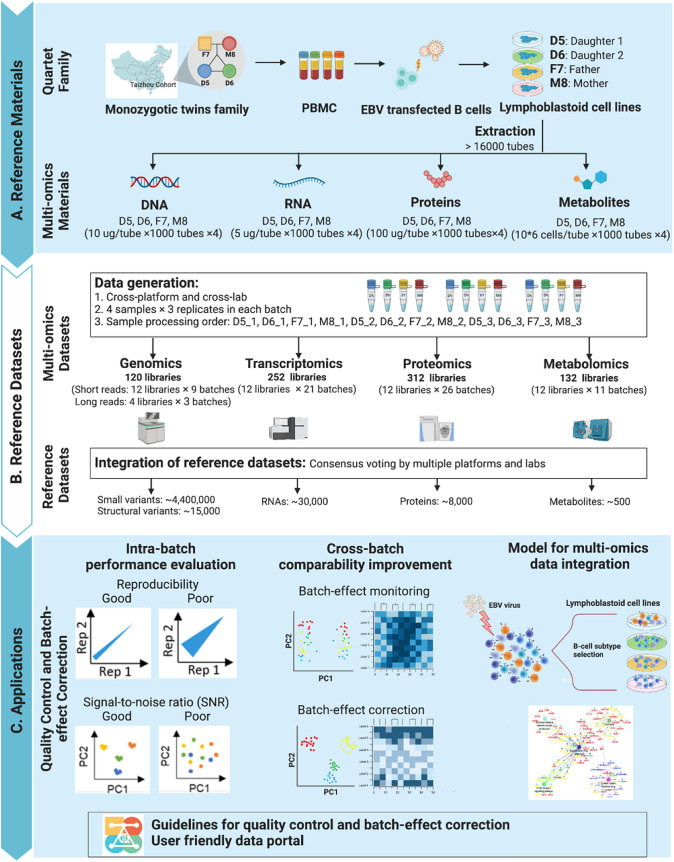
The quartet project for quality control of multiomics profiling

The Quartet Project, aiming at quality control and data integration of multiomics profiling, has established a series of openly consented multiomics reference materials, including matched DNA, RNA, protein and metabolite, derived from the same batch of immortalized EBV infected B-lymphoblastoid cell lines from a healthy Chinese Quartet family with parents and monozygotic twin daughters (Fig. 1) (Zheng et al. 2023). Replicates of each Quartet reference material were analyzed in each batch for performance evaluation. Correctly classifying different Quartet samples based on multiomics features can be used for assessing the reliability of correlation-based multiomics network integration. The subtle known biological differences among the four reference samples may allow technical biases and batch effects to be discerned more efficiently when using multiple sample reference materials.

Recently, NIST has partnered with institutions around the world to form an open consortium, the International Microbiome and Multi-Omics Standards Alliance (IMMSA), to address multiomics measurement challenges for microbiome. The IMMSA has five working groups planning to develop microbial reference materials, benchmark bioinformatic tools, establish best practices for metabolomics, develop standards for documents and written, and develop standard methods for enumerating whole-cell reference materials. Human whole stool is one source of their candidate reference materials, because it can facilitate the understanding of biologically relevant properties of the human gut microbiome and identify new biomarkers that may serve as disease indicators.

## Reference Datasets for Reference Materials

Establishing reference datasets for biological reference materials is crucial for evaluating the performance of high-throughput technologies. These reference datasets behave like "examination papers with right answers" to identify false-positive and false-negative results in a given test. Reference datasets can be qualitative, which involves identifying the presence or absence of variants, transcripts, proteins, or metabolites in the sample. They can also be quantitative, which involves determining the concentration, abundance, or expression levels of these molecules. Since a single run of a sample can lead to errors or miss true analytes (substances), reference datasets need to be carefully established by integrating data from various measurement technologies and bioinformatic analysis pipelines to avoid biases toward a specific method or platform. This ensures that the reference datasets provide an unbiased ground truth for evaluating the performance of multiomics technologies.

### Qualitative Reference Datasets

Reference datasets of variants for DNA reference materials typically include two components: benchmark variants and benchmark regions. Benchmark variants are a group of well-characterized and highly confident variants on the genomic sequences of a DNA reference material. These variants are developed with corresponding benchmark regions that include positions of the benchmark variants and homozygous reference positions.

To ensure the accuracy of benchmark variants, four approaches are commonly employed. First, benchmark variants are developed by integrating data from multiple sequencing technologies and bioinformatic algorithms to take advantage of the strengths of different methods, while carefully filtering out errors introduced from individual runs. To increase confidence in the benchmark variants, majority voting methods are often applied to select variants that are consistent among replicates. However, it is important to note that even reproducible variants may not always be true variants, as systematic errors shared across multiple methods can also be present (Robasky et al. 2014). Consensus genotype calls or in silico datasets can also be used to train machine learning models to find the optimal classification threshold to identify likely false positives. For example, the GIAB consortium used concordant genotype calls to train a simple one-class model for each dataset to determine whether each call from each dataset might be biased (Zook et al. 2019). Another example is the SEQC2 study, which spiked in silico SNVs and indels into normal replicates using BAMSurgeon to create "pseudo-tumors" (Fang et al. 2021). Variants detected by virtual tumor-normal pairs that were not spiked in were labeled as false positives. About 100 genomic and sequencing features were extracted to train adaptively boosted classifiers, which were used to classify variants called from real tumor-normal pairs into four confidence levels.

Second, pedigree information can be used to remove technical errors when establishing reference datasets for germline variants. Since the number of Mendelian inconsistent variants is far more than that of de novo variants and somatic variants arisen somatically or from cell culture, they are potential technical artifacts (Conrad et al. 2011). Illumina released another version of small-variant benchmark calls for NA12878—“the Platinum Genome” (Eberle et al. 2017). Although the Platinum Genome was integrated from sequencing datasets generated from a single Illumina sequencing platform and called by multiple pipelines, its accuracy was validated by using haplotype inheritance information though a well-studied 17-member pedigree. Benchmark variants of the Quartet samples are required to be the same between the monozygotic twin daughters and follow Mendelian inheritance law with parents.

Third, validation of draft benchmark variants using orthogonal technologies with different principles is necessary to confirm their reliability. Sanger sequencing is widely recognized as the "gold standard" method for validating variant calls from high-throughput sequencing. Additionally, array-based genotyping and amplicon-based sequencing can be designed to validate variants in specific regions of interest. Given the impracticality of validating millions of variants across the entire human genome simultaneously, a small number of variants with different confidence levels are randomly selected for validation. Variants with the highest confidence level are expected to fully supported by orthogonal technologies, and validation rate drops as the confidence level decreases (Fang et al. 2021). An alternative way is to focus on validating suspicious false positives. It should be noticed that discrepancies by orthogonal technologies do not necessarily indicate errors in benchmark variants, because variants on repetitive regions are unlikely to be easily characterized by sequencing technologies mentioned above, and long-read sequencing are more useful for such variants.

Fourth, manual inspection is necessary on discrepancies between benchmark variants and orthogonal validation. To ensure the accuracy and completeness of the benchmark sets, the GIAB consortium has established a process that involves sharing a draft benchmark with GIAB after initial evaluations at NIST, inviting volunteers with expertise in different technologies to contribute callsets, comparing these callsets to the draft benchmark, and randomly selecting putative false positives and false negatives for curation by the callset contributors (Wagner et al. 2022). Any sites identified as questionable or errors in the benchmark are re-curated by NIST. If the majority of false positives or false negatives are found to be errors or questionable in the benchmark, a new version of the benchmark is developed. This process ensures the continuous improvement and refinement of the benchmark sets, leading to more reliable and accurate variant calls.

Benchmark regions, also known as high-confidence genomic regions, are areas where accurate genotypes can be reliably derived, and sites within them are either benchmark calls or homozygous reference calls. Benchmark regions are often integrated from callable regions of multiple sequencing datasets that have relatively high mapping quality and sequencing coverage. Low complexity regions and highly repetitive regions are typically excluded to avoid possible systematic mapping errors, and flanking regions of uncertain variants are also excluded. Concordant variants outside of the high-confidence regions are not considered as benchmark variants due to their lower confidence level. Benchmark regions can be continually updated with advances in sequencing technologies, assembly algorithms, and variant calling methods. Linked-read and long-read sequencing technologies have been utilized to provide better coverage of difficult-to-map and repetitive regions. The benchmark regions for small variants have been expanded from 77% to 92% of the autosomes and X chromosome of GRCh38 by including long reads (Chin et al. 2020; Wagner et al. 2022). GIAB has collaborated with the Human Pangenome Reference Consortium (HPRC) and the Telomere-to-Telomere Consortium (T2T) to expand benchmark sets by utilizing T2T assemblies of the HG002 genome, initially focusing on chromosomes X and Y and later expanding to the entire genome (Nurk et al. 2022).

Identification of transcripts, proteins and metabolites is a critical step in interpreting omics datasets. In fact, the number of identified features (protein or metabolites) has been routinely used as a performance metric. As we have no idea what substances are expected to be present in biological reference materials, features are retained in the reference datasets if they are consistently detected among multiple replicates, platforms and algorithms with small coefficient of variation (CV) of quantitative measurements (Aristizabal-Henao et al. 2021; Davis et al. 2019). For reference materials with two or more sample groups, the number and identities of differentially expressed features between sample pairs are additional qualitative properties for quantitative omics.

### Quantitative Reference Datasets

Quantitative properties fall into two categories: absolute and relative quantification. Absolute quantification is to determine absolute copy numbers of transcripts or absolute concentrations of proteins and metabolites, which can be achieved by using a standard curve created by dilution series of internal standards for each substance. In the MAQC study, the expression levels of 1044 genes in samples A and B were measured by TaqMan qPCR assays, which were later used as orthogonal gold standard to assess the accuracy of microarray and RNA-seq (MAQC Consortium 2006, 2014). In the Quartet study, absolute quantification of Quartet protein reference materials was performed by using C^13^ stable isotope-labeled concatenated peptides. They randomly selected 33 proteins with intensity-based absolute quantification values distributed in four orders of magnitude as anchor proteins for absolute quantification and calibration of the copy numbers for the whole proteome. The abundance of more than 4000 proteins in each of four Quartet samples were quantified by aligning to the anchor proteins (Tian et al. 2023). NIST reported mass fraction, mass concentration and amount-of-substance concentration of metabolites for SRM 1950 by combining different extraction procedures, analytical methods, chromatographic separations and LC detection modes (Simon-Manso et al. 2013).

Absolute quantification is difficult to achieve in RNA-seq and untargeted metabolomics and proteomics. The original signals from a single sample, such as fragments per kilobase of transcript per million mapped reads (FPKM) in transcriptomics, fraction of total (FOT) in MS-based proteomics and relative peak areas in metabolomics, from different platforms, labs and batches are incomparable due to technical biases and batch effects (Zheng et al. 2023). These technologies are usually applied to compare expression profiles between control and test groups to identify differentially expressed features. Reference datasets of relative quantification, which determines fold changes in expression levels between different sample groups, is vital for performance assessment of such technologies (MAQC Consortium 2006, 2014). Recently, the Quartet Project established the first ratio-based quantitative reference datasets for transcriptomics, proteomics and metabolomics, through converting the original signals to relative quantitative measurements by dividing the expression profiles of study samples by those of a universal reference material on a feature-by-feature basis (Zheng et al. 2023).

## Performance Metrics

**Fig. 2:**
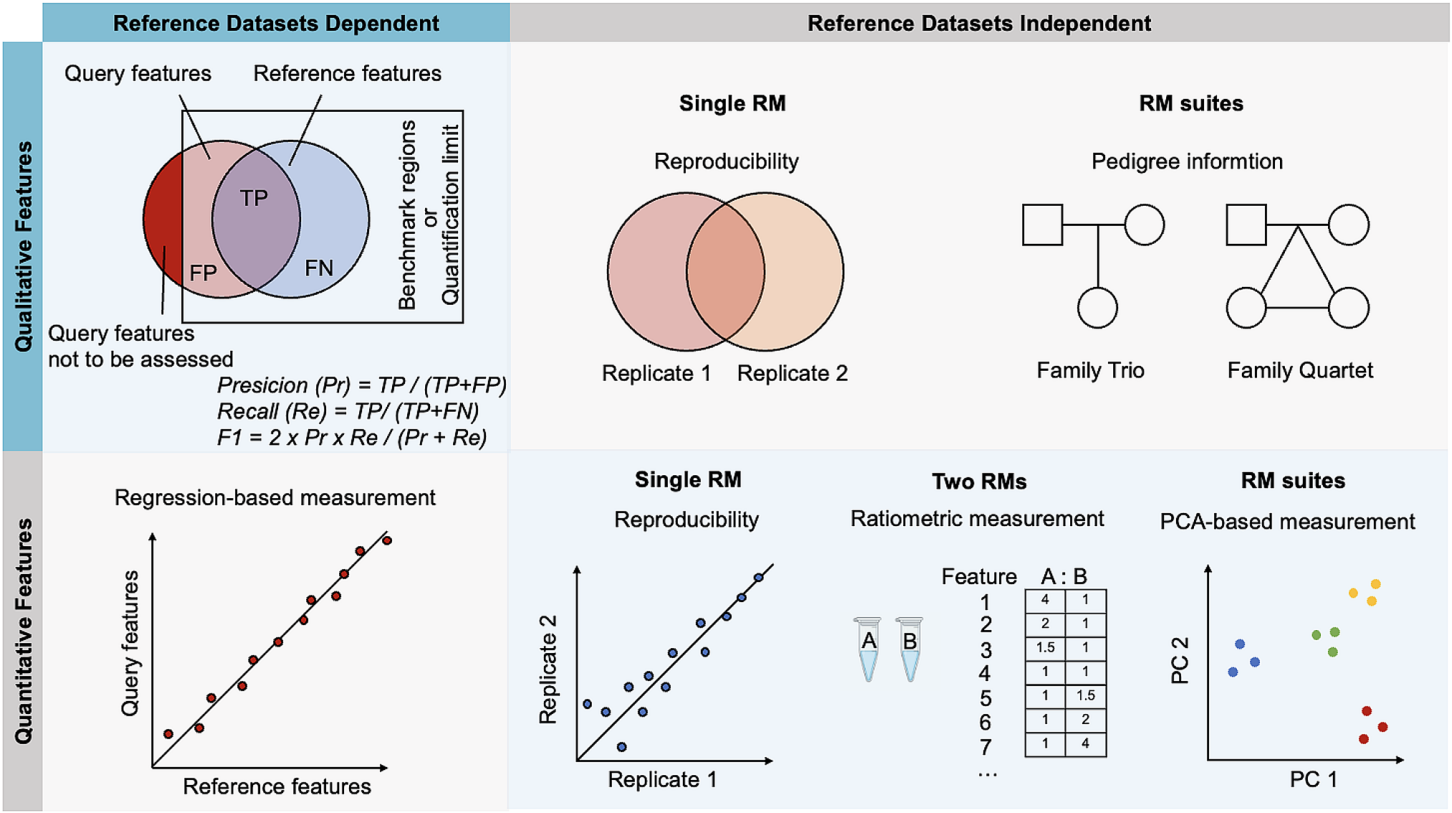
Performance metrics based on reference materials in multiomics profiling. When reference datasets are available, the performance of a query dataset can be assessed by comparing its consistency with the reference datasets. In cases where reference datasets are not available, the performance of a query dataset can be assessed by evaluating the reproducibility between replicates or utilizing built-in truth within multi-sample reference materials

Performance metrics are essential for evaluating the quality of a test dataset or experiment. These metrics are categorized into two groups: reference dataset-dependent metrics and reference dataset-independent metrics (Fig. 2). Reference dataset-dependent metrics assess the performance of variant calls and expression profiles by comparing them to a reference dataset. On the other hand, reference dataset-independent metrics evaluate the performance of the experiment without using a reference dataset, usually by the reproducibility of replicates or built-in truth of multi-sample reference materials.

### Reference Datasets Dependent

The evaluation of variant calls can be accomplished by comparing them to benchmark variants in benchmark regions. Two commonly used measures are precision, which refers to the fraction of called variants that are also benchmark variants, and recall or sensitivity, which is the fraction of benchmark variants that have been detected. Precision and recall are often in a trade-off relationship; improving one measure may result in a decrease in the other. The recall rate is not affected by false positives and can be increased by relaxing variant filtration thresholds, which may lead to detecting more putative variants. Therefore, it is necessary to assess both precision and recall to avoid over-inflating the recall rate. The F1-score is a commonly used measure that takes into account both false positives and false negatives by computing a harmonic mean of precision and recall. Specificity, which describes how many of all homozygous reference sites were correctly detected as non-variant sites, is less useful for variant calling performance evaluation because there is typical a three-orders-of-magnitude difference in the number of variants and the number of bases in a genomic region.

Benchmark variants developed from existing technologies and platforms are often limited to more easily detected variants and regions, which may lead to overestimating the overall performance of an assay when it is used to call variants outside of the high-confidence or benchmark regions. Variants outside of benchmark calls are often located in complex genomic regions and are less concordant between different callsets. In particular, the precision of an assay may be greatly inflated by using only benchmark regions.

As a variant or group of variants may be differently represented between variant call format (VCF) files, variant representation should be normalized before benchmarking to greatly reduce ambiguities. The Global Alliance for Genomics and Health (GA4GH) Benchmarking Team and GIAB have developed best practices and methods for benchmarking small germline variant calls (Kumaran et al. 2019), by providing guidance to match variant calls with different representations, define standard performance metrics, and stratify performance by variant type and genome context.

Reference datasets of transcriptomics, proteomics and metabolomics consist of a catalog of highly confident features or differentially expressed features (qualitative properties) and their corresponding expression levels or fold changes between sample groups (quantitative properties). Precision and recall are used to describe the proportion of detected features that are true and the proportion of reference features that are detected, respectively. Instead of F1-score, Matthews correlation coefficient (MCC) was employed as the main evaluation measure in the MAQC projects, because it takes true negatives into consideration and produces a high score only if good results are obtained in all of the four categories in a confusion matrix (Chicco and Jurman 2020). The Pearson correlation coefficient between the expression levels or fold changes of test datasets and reference datasets is used to describe the accuracy of quantitation. The root mean squared error (RMSE) measures the deviation between predicted and actual expression values. It should be noticed that reference datasets of biological reference materials may be incomplete, as features can be removed if their expression levels are below the limit of quantification, or they can only be measured by specific extraction and analysis procedures that have not been included in the construction of the reference datasets.

### Reference Datasets Independent

**Fig. 3:**
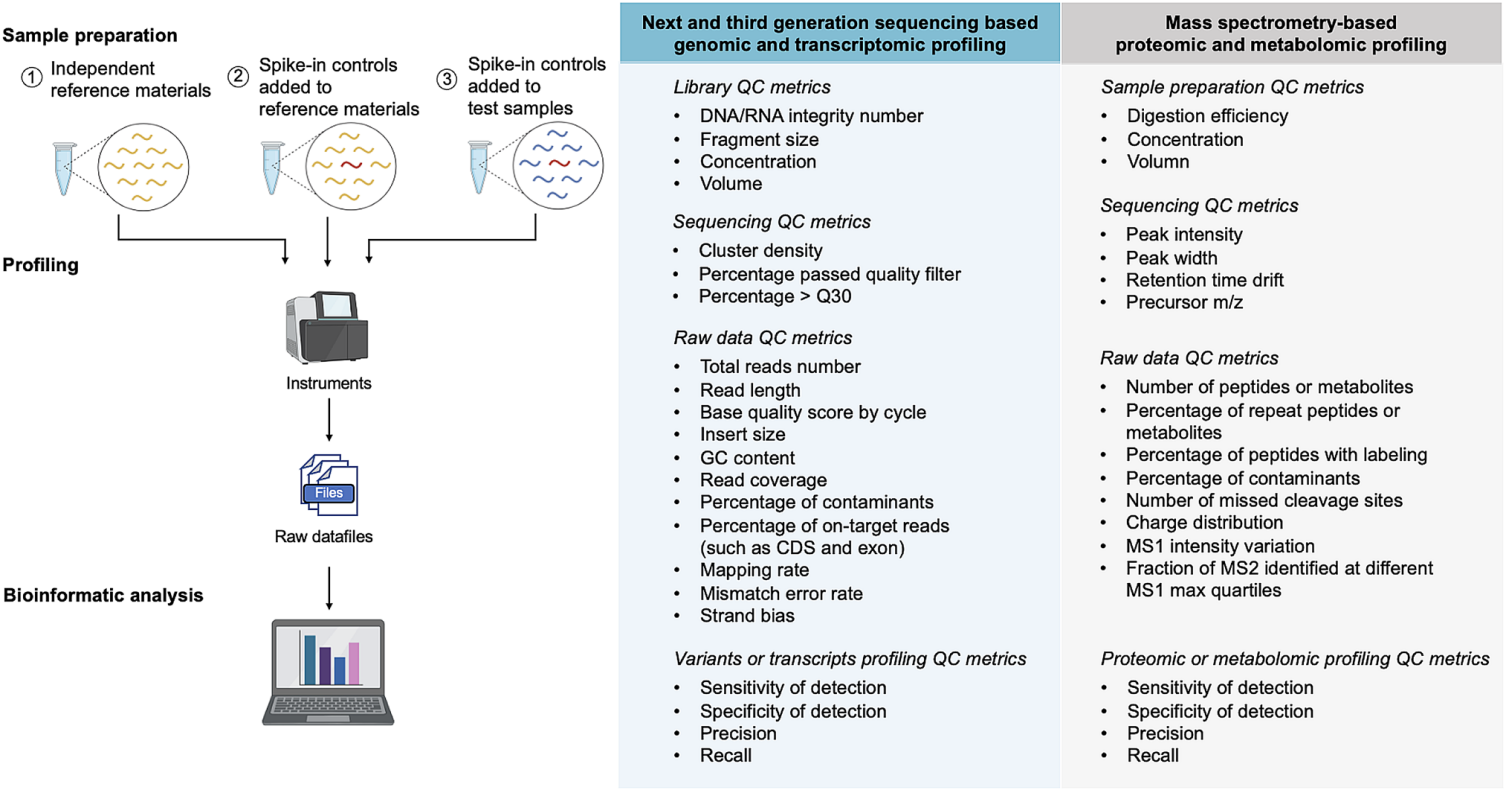
Schematic overview of multiomics profiling and quality control workflows showing the use of reference materials. Omics reference materials can evaluate all steps of sequencing workflow, including sample or library preparation, sequencing, raw reads, and profiling. Illustrated is the workflow of sequencing and key performance metrics for each step

To diagnose the underlying causes of suboptimal datasets, it is necessary to combine performance metrics for various stages of the experiment including sample or library preparation, sequencing, raw datasets, and variant detection (Fig. 3). This approach can help identify the specific areas where improvements are needed to enhance the quality of the data. High-quality sequencing data improve the quality and comparability of profiling results. Confirming the quality of sequencing libraries before committing them to sequencing runs increases the chances of success. In next- and third generation sequencing-based genomics and transcriptomics, important performance metrics for run-time sequencing quality include the number of reads or bases produced in the run, the percentage of bases called incorrectly at any one cycle, the percentage of bases with a base quality score, cluster density on the flow cell, and library complexity (Patel and Jain 2012). Raw read quality control assessments help filter out low-quality reads and trim low-quality bases at both ends of a read. After the mapping of preprocessed reads, sequencing alignment performance metrics can be used to assist in detecting biases in sequencing and mapping processes. In MS-based proteomics and metabolomics, the quality of datasets is greatly affected by instrument performance. The better the instrument performance, the better the data. Key performance metrics of the instrument extracted from raw datasets include electrospray stability, cleanliness of the ion source, cleanliness of the inner components of the MS, fragmentation efficiency, and mass accuracy (Morgenstern et al. 2021). For example, the uniformity of MS1 intensity distribution reflects the consistency of chromatographic spray and mass spectrometry sensitivity, while the uniformity of MS2 intensity distribution reflects the consistency of fragment ion detection sensitivity. There is no universally accepted standard for pre-analytical performance metrics. Appropriate thresholds depend on specific library preparation protocols, sequencers or instruments, and algorithms. While these metrics can be calculated for samples of interest, the use of widely adopted reference materials facilitates better understanding of performance across different assays and laboratories.

In cases where reference datasets are either unavailable or do not contain the features of interest, alternative methods can be employed to assess the performance. One such method involves evaluating the reproducibility of replicates, which compares the results of multiple measurements conducted on the same sample. Another approach is to utilize built-in truth from multi-sample reference materials, whereby a known standard is employed to evaluate the accuracy of the experiment.

The performance of variants calling results can be assessed by the repeatability and reproducibility of technical replicates or the Mendelian consistent ratio of family members. Technical replicates share the same variant calls and de novo mutations are rare; therefore, the majority of discordant variants is likely to represent genotyping errors (Veltman and Brunner 2012). The advantage of those reference datasets independent metrics is that they can evaluate the precision of variant calling on the whole genome without being restricted to the benchmark regions. However, these metrics cannot indicate how many true variants should be identified, or what the recall rate is.

To assess the accuracy of quantitative omics, three levels of reference dataset-independent metrics can be employed based on the number of available reference materials (Fig. 2). If a single reference material is available, the reproducibility between technical replicates is used to assess the performance of profiling results. However, a high correlation between two replicates of the same sample is not enough to ensure to accuracy in detecting differences between sample groups, because the replicates may share the same technical biases. If a pair of RMs is available, fold changes of features between sample pairs are expected to be the same as the designed expression signal ratios. If three or more RMs are available in a suite, PCA-based metrics can be used to assess the performance of distinguishing the intrinsic biological differences between sample groups.

## Utilization of Reference Materials

**Fig. 4:**
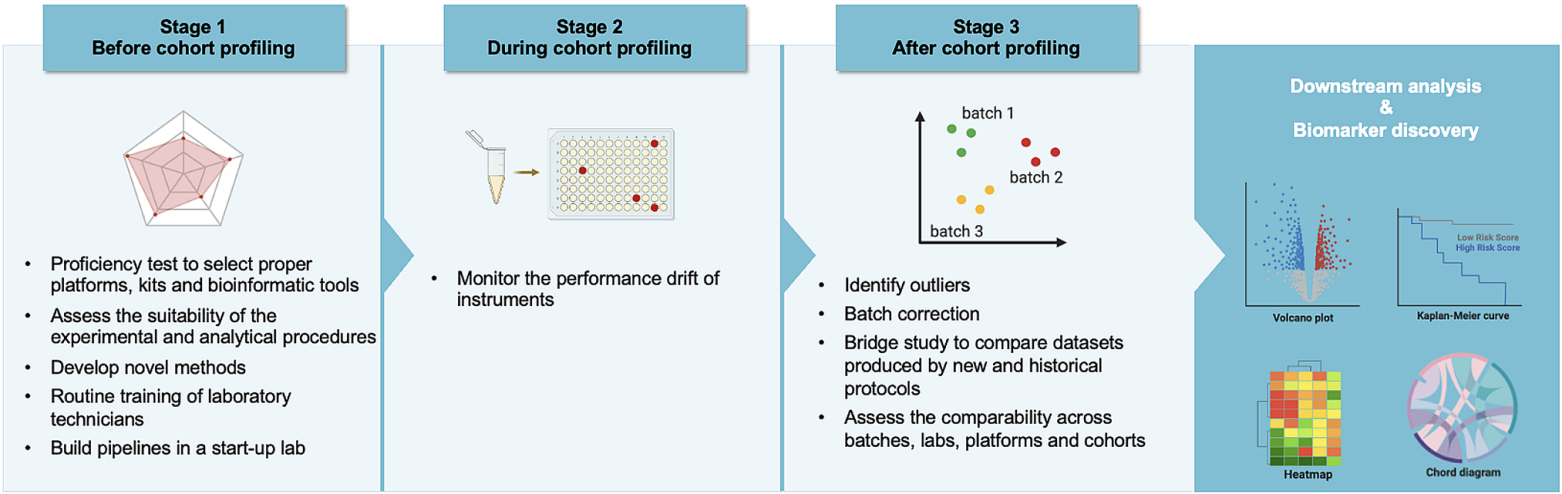
Utilization of reference materials in large cohort study to enhance the rigor and reproducibility of biomarker discovery

Identifying reliable biomarkers that can accurately predict disease risk or response to treatment is a critical goal of omics-based cohort studies. Large cohort studies that involve collecting samples over a long period of time and profiling the samples with multiple platforms at multiple labs may suffer from issues related to data incomparability and batch effects, which add difficulties for biomarker discovery. In this section, we discuss how omics RMs can be integrated in large cohort studies to enhance the rigor and reproducibility of biomarker discovery (Fig. 4).

To ensure accurate and reliable results from large-scale analysis of precious cohort samples, it is important to assess the suitability of experimental and analytical pipelines using reference materials prior to initiating the data generation process. The first important step is to choose the suitable RMs based on study design and instruments available. Points of consideration include the availability of RMs, their comparability to the test material, and whether the assigned property values and their confidence levels include the features of interest. The matrix composition of RMs is a critical consideration in the QC process of LC–MS. The performance indicators, such as calibration effectiveness, extraction efficiency, column performance, and ion suppression level, are directly influenced by the composition of the sample matrix. To ensure accurate and reliable performance assessment, it is recommended to employ RMs with a matrix composition as similar as possible to that of the study samples.

At each omics level, a variety of sample preparation methods, data generation platforms, and bioinformatic tools are available. By utilizing RMs in benchmark studies and proficiency test, researchers can gain insights into the strengths and limitations of various methods and technologies. This knowledge facilitates the selection of appropriate experimental and analytical procedures tailored to the specific goals, samples types, and available resources.

RMs can also be effectively used to optimize protocols and parameters by identifying and troubleshooting potential issues. For example, in genomics and transcriptomics by NGS, sequencing performance is influenced by the insert fragment size, which is associated with DNA shearing time. Longer shearing time produces shorter DNA fragments, and the insert fragment sizes must be measured to ensure that they fall within the expected molecular weight range (Fang et al. 2021). In MS-based proteomics and metabolomics, RMs can be used to assess mass-to-charge (m/z) ratio and chromatographic characteristics such as retention time, peak area, and peak shape, by comparing them to predefined acceptance criteria (Nakayasu et al. 2021). If the acceptance criteria are not met, corrective maintenance of the instruments or verification of reagents should be performed until the system suitability meets the requirements. Furthermore, reference materials can be used to train laboratory technicians to perform optimally in daily practice.

When conducting large-scale profiling of cohort samples, it's important to incorporate RMs into the experimental design to objectively monitor and evaluate the longitudinal stability of instruments and assays. In MS-based proteomic and metabolomic studies, RMs are typically run before and after a block of samples to monitor instrument performance drift and to ensure optimal settings are being used (Bittremieux et al. 2018). The block size is determined based on the expected performance drift over time and the separation length. In genomics and transcriptomics, DNA and RNA reference materials can be added to each batch of samples to be sequenced (Ren et al. 2023; Yu et al. 2023). This helps monitor the stability and comparability of analytical instruments across different batches, assays, and labs.

After large-scale profiling, datasets of both cohort samples and RMs are obtained. Datasets from the same RMs can reveal batch effects across labs, platforms, and time points. To eliminate batch effects, cohort sample datasets can be aligned to a common RM, removing unwanted variation and increasing comparability and statistical power, leading to greater confidence in biological insights from combined datasets of multiple batches. Sometimes, large-scale studies take a long time to complete and sequencing technologies can be updated, or new cohorts are needed to address important scientific questions. In such cases, bridge studies can be performed to compare the comparability of novel and historical protocols by using common RMs in both studies.

To ensure quality control in quantitative omics studies, the utilization of multi-sample RMs is essential, especially when investigating differences between cases and controls or various disease subtypes. In most proteomic and metabolomic studies, the reproducibility within technical replicates of a single reference material is commonly employed to evaluate dataset quality. Assessing the reproducibility of RMs is crucial for determining the stability and precision of an analytical method. However, relying solely on reproducibility may not be sufficient for accurately identifying biological differences between sample groups, as technical biases can impact the absolute abundance of measurements without affecting the relative differences between sample groups (Yu et al. 2023; Zheng et al. 2023). To achieve statistical significance, at least three replicates for each RM are necessary. In a PCA plot, a high-quality dataset is expected to show both separation between sample groups and tight clustering of replicates from the same sample. This indicates that the technical variation is under control and that the biological differences between sample groups are significant. In contrast, a low-quality dataset may show overlapping or scattered sample groups, indicating a high level of technical variation or noise that could obscure the biological differences between the groups.

## Challenges and Future Directions

High-throughput profiling technologies have revolutionized omics studies by enabling the generation of vast amounts of data in a relatively short period of time, allowing researchers to comprehensively study complex biological systems at an unprecedented level of resolution. However, performing high-throughput profiling is a highly complex and challenging process, and there are many potential sources of variability that can impact the results and reproducibility. Therefore, rigorous QA/QC is crucial to ensure confidence in the resulting data and biological discoveries. The use of RMs is an important aspect of QA/QC in high-throughput technologies to ensure accurate and reliable results. In this review, we aim to offer a comprehensive overview of the significance of utilizing well-characterized RMs across different levels of omics research, including genomics, transcriptomics, proteomics, and metabolomics. We provide insights into the characteristics, advantages, and limitations of RMs in each omics field, which are summarized in Table 2. Our goal is to assist researchers in making informed decisions when selecting suitable RMs for their specific research questions and analytical methods. Ultimately, the utilization of appropriate RMs can greatly enhance the accuracy and reliability of omics research outcomes.

**Table 2:** Characteristics of genomic, transcriptomic, proteomic, and metabolomic reference materials

Genomic reference materials			
Characteristics	Biological	Engineered	Synthetic
Source	Genomic DNA from cancer cell lines or lymphoblastoid cell lines	Genomic DNA of cell lines with specific genomic variants engineered at predetermined positions	Artificially generated DNA sequences that are designed to mimic specific genomic regions or variants but cannot be aligned to human reference genome
Intended usage	Performance assessment of variant calling from whole-genome, whole-exome and targeted sequencing	Performance assessment of genetic testing	Spiked into study samples as internal controls; performance assessment of quantitative genomic features (VAF, copy number, etc.)
Reference datasets	Millions of benchmark variants are integrated from multiple platforms and bioinformatic pipelines, but comprehensive characterization on whole genome is challenging	Limited number of engineered variants	Known DNA sequences and must establish commutability with biological samples before use
Representativeness	Readily commutable with biological samples, but not representative of population genetic diversity or tumor genome heterogeneity	Readily commutable with patient samples	May not fully represent the complexity of natural genomes
Preparation	Genomic instability and drift in cell lines can lead to lot-to-lot variability	Can cause off-target mutations and unintended effects	Represent any variant or sequence of interest, limited only by the constraints of synthesis
Consent and privacy considerations	Yes	No	No

Transcriptomic reference materials		
Characteristics	Biological	Synthetic
Source	RNA from natural sources, such as tissues, cell lines, etc.	Artificially generated RNA sequences
Intended usage	Performance assessment of differential gene expression from RNA sequencing, microarray, ddPCR and qPCR	Spiked into study samples as internal controls Performance assessment of differential gene expression, sensitivity (lower LOD) and dynamic range from RNA sequencing
Reference datasets	Fold changes of differential expressed genes integrated from multiple technologies, platforms and labs Gene with relatively low expression may not be included	Known RNA sequences with no homology to natural reference genomes Defined fold changes between two synthetic controls
Representativeness	Reflect the complexity and diversity of gene-expression patterns observed in biological samples, but do not fully capture the entire spectrum of transcriptomic variations and complexities present in different biological contexts	May not fully capture the complexity and biological context present in a natural system
Preparation	RNA exhibits variations across batches, even derived from the same cell lines RNA is prone to degradation	Synthesized with high precision and homogeneity across batches
Consent and privacy considerations	Yes	No

Proteomic reference materials		
Characteristics	Biological	Synthetic
Source	Proteins from natural sources, such as cell lysates, tissues, or bodily fluids	Purified proteins or chemical synthetic peptides
Intended usage	Performance assessment of identification and quantification for proteins	Spiked into study samples as internal controls Performance assessment of identification and quantification for proteins
Reference datasets	The complete proteome may not be fully characterized, only high confident proteins are identified by multiple platforms and techniques, with information of the identity and concentrations of proteins	Specific protein targets with defined and known composition, including the identity and concentration of the protein analytes
Representativeness	Reflect the complexity and diversity of the proteome in biological systems, but do not capture the entire rage of proteomic variations and complexities observed in different biological contexts	May not fully capture the complexity and biological context present in a natural system
Preparation	Proteins exhibit variations across batches, even derived from the same cell lines Protein reference materials prepared as peptides cannot assess the performance of digestion methods	Synthesized with high precision and homogeneity across batches
Consent and privacy considerations	No	No

Metabolomic reference materials		
Characteristics	Biological	Synthetic
Source	Metabolites from natural sources, such as tissues, cells, bodily fluids or microbial cultures	Chemical synthetic metabolites or isotopically labeled metabolites
Intended usage	Performance assessment of identification and quantification for metabolites	Spiked into study samples as internal controls Performance assessment of identification and quantification for metabolites
Reference datasets	The complete metabolome may not be fully characterized, only high confident metabolites are identified by multiple platforms and techniques, with the information of the identity and concentrations of metabolites	Specific metabolites with defined and known composition and concentration
Representativeness	Represent snapshot of the metabolites present in a specific biological system, but do not capture the entire rage of metabolome variations and complexities observed in different biological contexts and conditions	May not fully capture the complexity and biological context present in a natural system
Preparation	Metabolites exhibit variations across batches, even derived from the same cell lines	Synthesized with high precision and homogeneity across batches
Consent and privacy considerations	No	No

By incorporating well-characterized RMs into omics research, researchers can overcome various challenges and limitations. RMs provide a standardized reference point that enables calibration and quality control throughout the experimental workflow. They serve as valuable tools for method optimization, validation, and troubleshooting, allowing researchers to assess the performance of their analytical methods and identify any potential biases or errors. Furthermore, the use of RMs facilitates inter-laboratory comparisons and promotes data harmonization, enabling the integration and comparison of results across different studies and platforms. Although the profiling of RMs may entail additional costs, implementing a thorough QA/QC methodology is important for evaluating and monitoring the performance of data generation processes. This upfront investment contributes to the long-term reliability and accuracy of the results, minimizing potential errors and ensuring the accuracy and reliability of the omics research.

The careful selection of RMs is crucial to ensure their relevance and applicability to the study at hand. Researchers should consider the intended use of the study and choose RMs that closely resemble the properties of the samples being investigated. Additionally, the selected RMs should be qualitatively and quantitatively representative of the entire collection of samples included in the study. This ensures that the RMs effectively mimic the characteristics of the biological samples, enabling accurate and meaningful comparisons and interpretations. When studying specific genetic or phenotypical features that vary among different ethnic groups, it is important to choose RMs that match the ethnicity of the study samples. This approach ensures that the RMs accurately reflect the characteristics of the study population, enabling the assessment of the detection performance of those specific genetic or phenotypical features (Hardwick et al. 2017).

As profiling methods continue to advance and new technologies emerge, the reference datasets for existing RMs will undergo continuous updates and refinements. One example of this is the utilization of long reads in genomic sequencing. Long reads are particularly valuable for profiling repetitive and complex regions, which are challenging to be mapped by short reads (Wenger et al. 2019). By incorporating long reads, benchmark variants in these regions can be better characterized (Wagner et al. 2022). Additionally, long-read technologies enable precise transcript detection and RNA modifications (Leger et al. 2021; Soneson et al. 2019). In proteomics, MS techniques are extensively used to study post-translational modification (PTMs) of proteins (Zecha et al. 2022). The reference materials will expand to encompass more omics types along with the development of technologies. For example, reference datasets of DNA epigenomics for DNA RMs can be developed, RNA RMs can include small RNA profiling and RNA modification reference datasets, and protein RMs can incorporate PTM reference datasets.

Challenges persist in the global promotion and adoption of reference materials and reference datasets. First, regulatory challenges, especially across different regions of the world, can pose additional obstacles in adopting a universal RM (Guerrier et al. 2012; Krogstad et al. 2010). Biological RMs, especially those intended for human genomics and transcriptomics, which are frequently derived from human specimens, require stricter adherence to informed consent principles and governmental controls. Currently, there is no single, comprehensive international model for governing human genetic resources. The distinct nature of informed consent across different countries, influenced by diverse cultures and social traditions, necessitated addressing legal, ethical, and logistical aspects related to genetic materials and data utilization while respecting each nation's sovereignty and cultural norms. International collaboration and agreements are imperative in addressing these challenges and ensuring the conscientious and equitable utilization of human genetic resources worldwide (Gainotti et al. 2016; van Belle et al. 2015).

Second, we strongly recommend that QC data should be made available alongside the study samples in databases or repositories that adhere to the FAIR principles (Findable, Accessible, Interoperable, and Reusable), which is crucial for enhancing data management and sharing (Conesa and Beck 2019; Wilkinson et al. 2016). Currently, QC information is often omitted from scientific publications, leading to uncertainty about the performance methodology used. In the future, guidelines may be developed to mandate the inclusion of QC metrics in data submissions to public repositories, similar to existing guidelines for other aspects of data reporting. Coupling comprehensive QC information to the experimental data will allow for quick assessment of the reliability of an experiment, which is crucial in light of recent reports of the general reproducibility crisis in various scientific fields (Anonymous 2021; Baker 2016; Shi et al. 2017). It is essential to prioritize and formalize QC practices to ensure the quality and reproducibility of high-throughput multiomics profiling results by fully utilizing well-characterized RMs and appropriate QC metrics.

## Conclusion

In this review, we summarized reference materials across all levels of omics, including (epi-)genomics, transcriptomics, proteomics, and metabolomics. We have offered a comprehensive overview of leveraging omics reference materials to enhance data quality. This initiative is geared toward promoting robust scientific research and advancing our understanding of complex biological systems through the thoughtful application of omics technologies.

## Data Availability

Not applicable.

## Abbreviations

ABRF: Association of Biomolecular Resource Facilities
ATCC: American Type Culture Collection
CDC: Centers for Disease Control and Prevention
CNA: Copy number alteration
CPTAC: Clinical Proteomic Tumor Analysis Consortium
CRM: Certified reference material
ctDNA: Circulating tumor DNA
EBV: Epstein–Barr virus
ERCC: External RNA Control Consortium
FFPE: Formalin-Fixed Paraffin-Embedded
gDNA: Genomic DNA
GeT-RM: Genetic Testing Reference Materials Coordination Program
GIAB: Genome in a Bottle Consortium
HPRC: Human Pangenome Reference Consortium
HUPO: Human Proteome Organization
IMMSA: International Microbiome and Multi-Omics Standards Alliance
JRC: Joint Research Centre
LDT: Laboratory developed tests
LOD: Limit of detect
LTR: Long-term reference
MAQC: MicroArray Quality Control Consortium
MCC: Matthews correlation coefficient
MetQual: Metabolomics Quality Assurance and Quality Control Program
MMR: Mismatch repair
mQACC: Metabolomics Quality Assurance and Quality Control Consortium
MRM: Multiple reaction monitoring
MS: Mass spectrometry
NCI: National Cancer Institute
NIM: National Institute of Metrology
NIST: National Institute of Standards and Technology
NMR: Nuclear magnetic resonance
PCA: Principal component analysis
PEA: Proximity Extension Assay
PRM: Parallel reaction monitoring
PSI: Proteomics Standards Initiative
PTM: Post-translational modification
QA: Quality assurance
QC: Quality control
RM: Reference material
RMSE: Root mean squared error
RT: Retention time
SEQC: Sequencing Quality Control Consortium
SNR: Signal-to-noise ratio
SNV: Single-nucleotide variations
SRM: Standard reference materials
SV: Structural variants
T2T: Telomere-to-Telomere
TCGA: The Cancer Genome Atlas
TMB: Tumor mutation burden
TNBC: Triple-negative breast cancer
UHRR: Agilent Universal Human Reference RNA material
VAF: Variant allele frequency
WES: Whole-exome sequencing
WGS: Whole-genome sequencing
